# Physical water scarcity metrics for monitoring progress towards SDG target 6.4: An evaluation of indicator 6.4.2 “Level of water stress”

**DOI:** 10.1016/j.scitotenv.2017.09.056

**Published:** 2018-02-01

**Authors:** D. Vanham, A.Y. Hoekstra, Y. Wada, F. Bouraoui, A. de Roo, M.M. Mekonnen, W.J. van de Bund, O. Batelaan, P. Pavelic, W.G.M. Bastiaanssen, M. Kummu, J. Rockström, J. Liu, B. Bisselink, P. Ronco, A. Pistocchi, G. Bidoglio

**Affiliations:** a European Commission, Joint Research Centre, Directorate for Sustainable Resources, Via E. Fermi 2749, 21027 Ispra (VA), Italy; b Twente Water Centre, University of Twente, P.O. Box 217, Enschede, Netherlands; c Institute of Water Policy, Lee Kuan Yew School of Public Policy, National University of Singapore, Singapore; d International Institute for Applied Systems Analysis, Laxenburg, Austria; e Faculty of Geosciences, Utrecht University, Utrecht, Netherlands; f Robert B. Daugherty Water for Food Global Institute, University of Nebraska, Lincoln, United States; g Flinders University of South Australia, National Centre for Groundwater Research and Training, College of Science and Engineering, Adelaide, Australia; h International Water Management Institute, Vientiane, Lao People's Democratic Republic; i Delft University of Technology, Stevinweg 1, 2600, GA, Delft, Netherlands; j UNESCO-IHE, Institute for Water Education, Westvest 7, 2611, AX, Delft, Netherlands; k Aalto University, Water and Development Research Group, Espoo, Finland; l Stockholm Resilience Centre, Stockholm University, Kräftriket 2b, 10691 Stockholm, Sweden; m School of Environmental Science and Engineering, South University of Science and Technology of China, Shenzhen, 518055, China

## Abstract

Target 6.4 of the recently adopted Sustainable Development Goals (SDGs) deals with the reduction of water scarcity. To monitor progress towards this target, two indicators are used: Indicator 6.4.1 measuring water use efficiency and 6.4.2 measuring the level of water stress (WS). This paper aims to identify whether the currently proposed indicator 6.4.2 considers the different elements that need to be accounted for in a WS indicator. WS indicators compare water use with water availability. We identify seven essential elements: 1) both gross and net water abstraction (or withdrawal) provide important information to understand WS; 2) WS indicators need to incorporate environmental flow requirements (EFR); 3) temporal and 4) spatial disaggregation is required in a WS assessment; 5) both renewable surface water and groundwater resources, including their interaction, need to be accounted for as renewable water availability; 6) alternative available water resources need to be accounted for as well, like fossil groundwater and desalinated water; 7) WS indicators need to account for water storage in reservoirs, water recycling and managed aquifer recharge. Indicator 6.4.2 considers many of these elements, but there is need for improvement. It is recommended that WS is measured based on net abstraction as well, in addition to currently only measuring WS based on gross abstraction. It does incorporate EFR. Temporal and spatial disaggregation is indeed defined as a goal in more advanced monitoring levels, in which it is also called for a differentiation between surface and groundwater resources. However, regarding element 6 and 7 there are some shortcomings for which we provide recommendations. In addition, indicator 6.4.2 is only one indicator, which monitors blue WS, but does not give information on green or green-blue water scarcity or on water quality. Within the SDG indicator framework, some of these topics are covered with other indicators.

## Highlights

• SDG target 6.4 aims at reducing water scarcity.
• Indicator 6.4.2 “Level of water stress”, relates water use to availability.
• We identify 7 key elements that need to be considered for a water stress indicator.
• Indicator 6.4.2 considers these 7 elements, but there is need for improvement.
• We give clear recommendations for improvement.

## Graphical abstract

**Figure:**
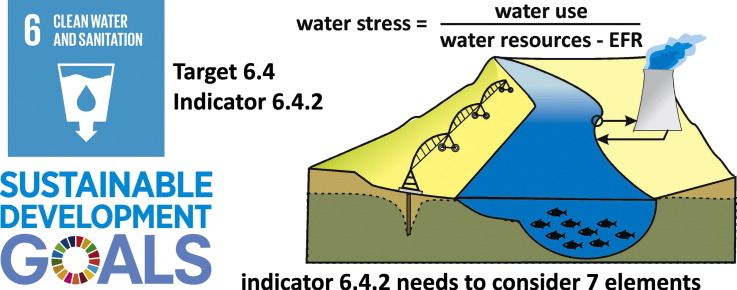

## 1 Introduction

Within the planetary boundaries framework, limited freshwater availability is identified as one of nine planetary boundaries (Steffen et al., 2015). Recently, Mekonnen and Hoekstra (2016) quantified that 4 billion people face severe water stress during at least one month per year, and 1.8 billion at least six months per year. Indeed, for providing the main three primary human needs of water, energy and food security, water is an essential resource for each (Vanham, 2016). Competition for it will grow due to increasing population, shifting lifestyles as well as climate change.

In September 2015, heads of state from around the world adopted the 2030 Agenda for Sustainable Development consisting of 17 Sustainable Development Goals (SDGs) and 169 targets. The 2030 Agenda includes a dedicated goal on water and sanitation (SDG 6), where target 6.4 deals with water scarcity (Table 1). In order to reach this target, two indicators are used: 6.4.1 and 6.4.2 (Table 1).

**Table 1:** SDG target 6.4 with relevant indicators, within SDG 6 “Clean water and sanitation”.

Target	Indicator
6.4: By 2030, substantially increase water-use efficiency across all sectors and ensure sustainable withdrawals and supply of freshwater to address water scarcity and substantially reduce the number of people suffering from water scarcity	6.4.1: Change in water-use efficiency over time
	6.4.2: Level of water stress: freshwater withdrawal as a proportion of available freshwater resources, computed as: (1) $\mathrm{WS}\;\left(\%\right)=\frac{\mathrm{WW}}{\left(\mathrm{TRWR}-\mathrm{EFR}\right)}\times 100$ with WS = water stress, WW = total freshwater withdrawn, TRWR = total renewable water resources, and EFR = environmental flow requirements

In the past, different water scarcity indicators have been developed (Liu et al., 2017, Rijsberman, 2006, Savenije, 2000). Physical water scarcity occurs when there is not enough water to meet all demands (including the environment). Blue water refers to liquid water in rivers, lakes, wetlands and aquifers (Rockström et al., 2009). According to Kummu et al. (2016), physical blue water scarcity can be fundamentally divided into two aspects: water shortage (population-driven water scarcity) and water stress (demand-driven water scarcity, i.e. the ratio water use to water availability) (Table 2). Water scarcity indicators also include economic or green water scarcity indicators (Table 2), where green water refers to rainwater held in the unsaturated zone of the soil and available to plants.

**Table 2:** Different water scarcity indicators.

Water scarcity indicators	Explanation
Physical blue water scarcity	Water shortage: refers to the impact of low water availability per person. Given a certain water endowment and per capita water requirement, water shortage can therefore be seen as population-driven scarcity.
	Water stress: refers to the impact of high water use (either withdrawals or consumption) relative to water availability. Stress can be seen as demand-driven scarcity, potentially occurring even when population is low, for instance because of large water-use for producing products for populations elsewhere. SDG indicator 6.4.2 is a water stress indicator
Economic water scarcity indicators	Economic water scarcity indicates where affordable water supply works are not available (Molden, 2007), thus showing where regions lack the necessary infrastructure to take water from rivers and aquifers.
Other indicators, e.g. green water scarcity indicators, combined blue-green water scarcity indicators	Following the definition of Rockström et al. (2009), green water is soil water held in the unsaturated zone, derived from precipitation and available to plants. Several green water scarcity indicators exist (Schyns et al., 2015b) as well as combined blue-green water scarcity indicators (Gerten et al., 2011, Kummu et al., 2014). Indicator 6.4.2 does not address green water scarcity. However, as the processes of origin of green and blue water are closely related (Savenije, 2000), we discuss this interaction in Section 5.2.

SDG indicator 6.4.2 is a blue water stress indicator, as it is defined as the ratio of total fresh water withdrawn by all sectors to the water availability (total renewable fresh water resources minus EFR) in a particular country or region (Table 1). The indicator neither addresses green water scarcity, nor economic water scarcity.

In this paper, our objective is to identify whether the currently proposed SDG indicator 6.4.2 considers the different elements that need to be accounted for in a water stress indicator. To do this, the following sections are included:

• In Section 2, we define the elements that need to be accounted for in a water stress indicator, which to our knowledge has not been bundled in the scientific literature in one paper before.
• In Section 3, we analyse the definition, concept and method of SDG indicator 6.4.2. We then analyse whether the elements as discussed in Section 2 are represented in SDG indicator 6.4.2, highlighting current shortcomings and recommendations for improvement
• In Section 4, we briefly discuss the proposed monitoring levels and related data availability
• In Section 5, we briefly discuss the water-stress related issues of water quality and blue-green water considerations

For clarity, Table 3 shows a list of the acronyms we use.

**Table 3:** Acronyms with definition.

Acronym	Definition
AQUASTAT	FAO's global water information system
CICES	Common International Classification of Ecosystem Services
EC	European Commission
EFR	Environmental flow requirements
ES	Ecosystem Services
FAO	Food and Agricultural Organisation of the United Nations
ISIC	International Standard Industrial Classification
IWMI	International Water Management Institute
MAR	Managed aquifer recharge
MDG	Millennium Development Goal
SDG	Sustainable Development Goal
TRWR; IRWR; ERWR	Total renewable freshwater resources; Internal renewable water resources; External renewable water resources
UN	United Nations
WEF nexus; WEFE nexus	Water-energy-food nexus; Water-energy-food-ecosystem nexus
WEI; WEI +	Water Exploitation Index; Water Exploitation Index +
WF; WFA	Water footprint; Water footprint assessment
WS	Water stress
WA	Water availability
WTA; CTA	Withdrawal-to-availability ratio; Consumption-to-availability ratio
WU	Water use
WWTP	Waste water treatment plant
WW	Water withdrawn

## 2 Elements to be considered when using or developing a particular WS indicator

### 2.1 Introduction

Most existing WS indicators compare water use (WU) with water availability (WA):

(2) WS=WU/WA

WU is generally measured as either gross or net water abstraction from fresh surface water or groundwater. WA is generally measured as the freshwater renewal rate, whereby sometimes an environmental flow requirement (EFR) (Tharme, 2003) is deducted. WS, WU and WA are generally estimated on annual or subannual, e.g. monthly, basis.

When gross water abstraction is used as indicator of WU, the resultant WS indicator is often called the withdrawal-to-availability ratio (WTA). When net water abstraction (also termed consumptive water use, water consumption or blue water footprint) is used as indicator of WU, the resultant WS indicator is also called the consumption-to-availability (CTA) ratio.

WTA and CTA are often demarcated by a threshold level, where values higher than 40% (or 0.4 when written as a fraction) denote “high WS” (Rockström et al., 2009). Based on earlier work of Balcerski (1964), Falkenmark and Gunnar (1974) and Szesztay (1970), Raskin et al. (1997) suggested that a country is severely water scarce if the ratio of annual withdrawal to annual renewable water resources exceeds 40%, water scarce if this ratio lies in the range of 20–40%, moderate water scarce when this ratio is in the range of 10–20%, and low water scarce when the ratio is below 10%. These values were adopted by the UN report “*Comprehensive assessment of the freshwater resources of the world”* (UN, 1997) and consequently widely used in the literature, e.g. Arnell (1999), Arnell (2004), Oki et al. (2001), Seckler et al. (1999) or Vörösmarty et al. (2000). Also the European Commission (EC) and the European Environmental Agency (EEA) use these threshold values in the Water Exploitation Index (WEI) (EEA, 2003), which takes gross water abstraction for water use, and the WEI + (Faergemann, 2012), which takes net water abstraction for water use.

Past global to regional WS studies have applied this relation (WU/WA) in different ways:

• The use of gross water abstraction (Arnell, 1999, Arnell, 2004, Vanham et al., 2009a, Vanham et al., 2009b), net water abstraction (Hoekstra et al., 2012, Kummu et al., 2016, Mekonnen and Hoekstra, 2016) or both (De Roo et al., 2016, Gawlik et al., 2017, Munia et al., 2016);
• The inclusion of EFR (Hoekstra et al., 2012, Liu et al., 2016, Mekonnen and Hoekstra, 2016, Vanham et al., 2009a, Vanham et al., 2009b, Wada et al., 2011) or not (Arnell, 1999, Arnell, 2004);
• Computing WS on an annual level (Vörösmarty et al., 2000) or monthly level (Hoekstra et al., 2012, Mekonnen and Hoekstra, 2016, Schyns and Hoekstra, 2014, Vanham et al., 2009b, Wada et al., 2011);
• Computing WS on country level (Seckler et al., 1999), catchment level (Hoekstra et al., 2012), or down to grid level (Mekonnen and Hoekstra, 2016);
• Specifically addressing (nonrenewable) groundwater resources (Chouchane et al., 2015, Gleeson et al., 2012, Schyns et al., 2015a, Schyns and Hoekstra, 2014, Wada et al., 2011) or not;
• Addressing other sources like desalination (Wada et al., 2011) or topics like water recycling.

Based on the above, we identify the following aspects as most relevant and discuss them further in this section:

1. Gross versus net water abstraction
2. Environmental flows (EF) or environmental flow requirements (EFR)
3. Temporal scale
4. Spatial resolution
5. Surface water and groundwater
6. Alternative water sources
7. Reservoirs, water recycling and managed aquifer recharge

### 2.2 Gross versus net water abstraction

**Fig. 1:**
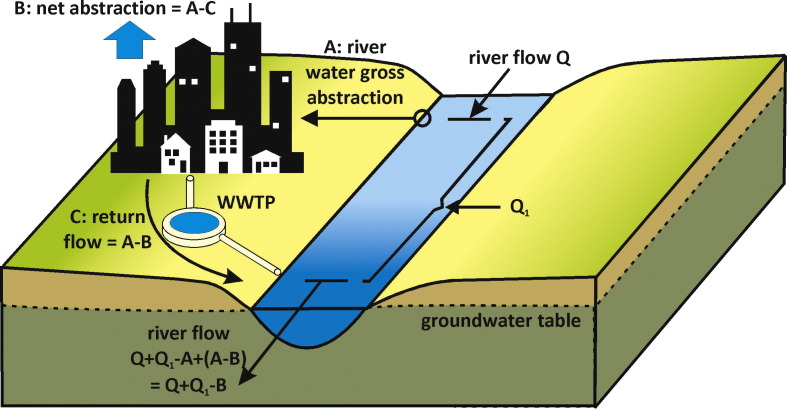
Simple representation of a river section where a city extracts its municipal water from (gross water abstraction A). Part from this water is “lost” from the river as consumptive water use B (net water abstraction) and part is returned (after treatment in a wastewater treatment plant or WWTP) downstream as return flow (A–B). Between the upstream gross water abstraction and downstream return flow, the river flow Q receives a hydrological surplus of Q_1_. This river section is however deprived of the quantity A (which makes a WS indicator using gross water abstraction relevant). Downstream of the return flow, the river is only deprived of the quantity B (which makes a WS indicator using net water consumption relevant).

We argue that both gross and net water abstraction can provide important information on WS and therefore can be used in a WS indicator. As an example, Fig. 1 shows that for certain river sections either gross or net water abstraction would give the most relevant information in a WS assessment.

**Fig. 2:**
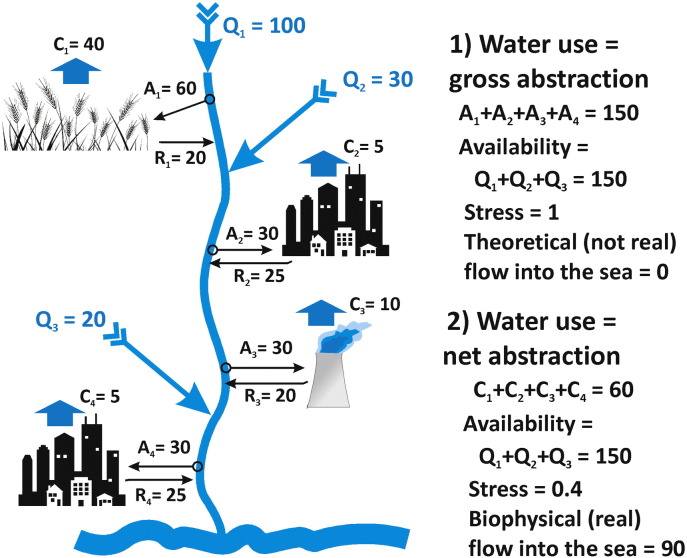
Difference in the calculation of WS (water use/water availability) when water use is defined as gross or net water abstraction, based upon a hypothetical case with a total water availability in the catchment of 150 units, a water use of 150 units (gross abstraction) or 60 units (net abstraction). Q = water availability; A = gross abstraction; C = net abstraction (consumption); R = return flow.

Fig. 2 shows for a small river basin a simple example of the difference in the calculation of WS (water use/water availability) when water use is defined as either gross or net water abstraction. In this particular case study, total water availability equals 150 units. The four water users (2 cities, 1 facility for energy production and 1 agricultural area with irrigation) use in total 150 units of water when we look at gross water abstractions, but 60 units of water when considering net water abstractions. WS therefore equals 1 when water use is taken as gross abstraction, resulting in a theoretical flow into the sea of 0 units (a closed basin). WS equals 0.4 when water use is taken as net abstraction, resulting in a flow into the sea of 90 units, which represents the actual biophysical situation for the whole basin, but underestimates WS for specific river sections between points of gross abstraction and return flow.

Industrial and agricultural products often have complex, spatially disconnected production chains. To quantify water use along a supply chain, the concepts of virtual water and water footprint (WF) have been introduced (Hoekstra and Mekonnen, 2012). Two different approaches to conduct a WF assessment exist in parallel and are published as the Water Footprint Assessment (WFA) Manual (Hoekstra et al., 2011) and the ISO 14046 document (ISO, 2014). Both approaches consist of an inventory stage and a sustainability or impact assessment stage, where WS is part of the sustainability or impact assessment phase. During the inventory phase, all consumptive water uses (net water abstractions) along the supply chain are quantified.

We summarize the following points:

• The use of gross and/or net water abstraction in a WS assessment depends on the scale and aim of the study. It is also possible to use both in parallel and compare results, as in the recent Urban Water Atlas of the European Commission by means of the WEI and WEI + (EC, 2017, Gawlik et al., 2017, UfM, 2017) and as done by De Roo et al. (2016) or Munia et al. (2016).
• The amount of gross abstracted water is for certain economic activities a determining factor. Certain components of public water supply require the full water abstracted, like water for showering or flushing a toilet. A large proportion of these gross water abstractions become return flows, only small fractions become consumptive water uses. Only accounting for net water abstraction in a WS assessment neglects this gross water requirement. Generally, about 10% of public gross water abstraction becomes consumptive use (Vanham and Bidoglio, 2014).
• Gross water abstraction is very relevant for groundwater, as rapid return flows like for surface water are generally not occurring for groundwater resources
• Data reliability: for domestic, industrial and energy use, net abstraction is often derived from gross abstraction statistics and therefore not as reliable as gross abstraction. On the other hand, modelling can quite reliably estimate blue water consumption for crops, whereas gross water abstraction data for irrigation are often lacking.
• When computing WS in supply chain analyses (water footprint assessment), net water abstraction is used

### 2.3 Environmental flow requirements (EFR)

It is now generally recognized that EFR need to be included in WS assessments. A widely used definition of environmental flow is “the quality, quantity, and timing of water flows required to maintain the components, functions, processes, and resilience of aquatic ecosystems which provide goods and services to people” (Hirji and Davis, 2009).

Water availability in WS assessments is expressed as the total renewable water resources (TRWR) minus EFR:

(3) Water availability=TRWR–EFR

**Fig. 3:**
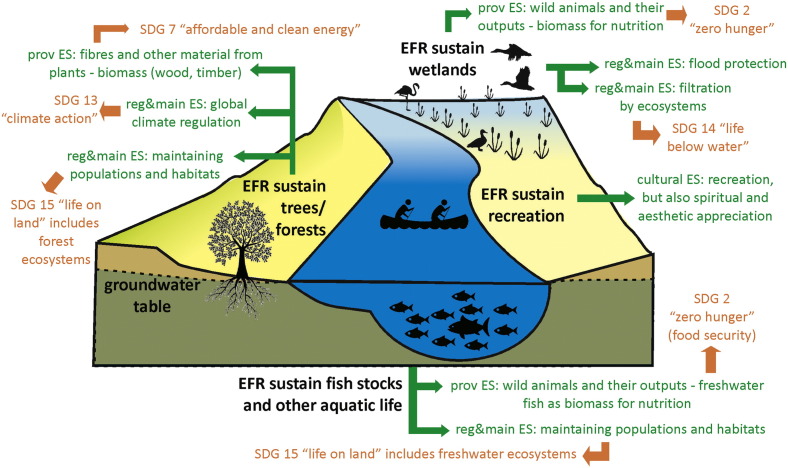
EFR sustain a list of ecosystem services (ES), of which some are displayed in the figure, with direct links to specific (non-exhaustive) SDG's. prov ES = provisioning ES; reg&main ES = regulating and maintaining ES. Definition of ES according to CICES (Common International Classification of Ecosystem Services) Version 4.3 (EEA, 2016).

This is also the way in which water availability is defined in SDG indicator 6.4.2. EFR sustain a wide range of ecosystem services (ES), which have direct links to specific SDG's (Fig. 3). For example, EFR sustain fish stocks and other aquatic life, which contribute as nutrition biomass directly to SDG 2 “zero hunger”. In certain rivers systems, like the Mekong, freshwater fish biomass contributes the bulk of animal protein intake of the regional basin population. With some 1700 species of fish, the Mekong is the second most aquatic biodiverse river basin in the world (Molle et al., 2010). EFR are a key requirement for maintaining freshwater populations and habitats (regulating and maintaining ES), thereby contributing to SDG 15 “life on land”, which includes the conservation of freshwater biodiversity. Two thirds of the lower Mekong basin's 55 million people are in some way active in fisheries, at least part-time or seasonally (Mather, 2009). Fisheries therefore contribute directly to SDG 8 “decent work and economic growth”.

Other ES sustained by EFR include the regulating and maintaining ES of natural flood protection by wetlands (Grizzetti et al., 2016). Wetlands and estuaries also provide the ES of filtration, which contributes to SDG 14 “life below water”, by reducing nutrient flows to downstream river sections and coastal zones. In addition, EFR sustain different cultural ES, like recreation but also aesthetic and spiritual appreciation. A well-known example of the latter is the Ganges in the Indian cultural setting (Lokgariwar et al., 2014).

Quantifying EFR is not straightforward because as a first step one has to decide what aspects of aquatic ecosystems or ecosystem services are to be protected. For the quantification of EFR, different methods have been identified (Falkenmark et al., 2007, Pastor et al., 2014, Richter et al., 2012, Smakhtin et al., 2004, Tharme, 2003), which can be grouped into three categories, namely hydrological, hydraulic-habitat and holistic methods (EC, 2015, Tharme, 2003). The simplest, typically desktop hydrological methods, primarily rely on the use of hydrological data, usually in the form of naturalized (pristine or naturalized river flow), historical monthly or daily flow records, for making EFR recommendations. These approaches are rapid, non-resource-intensive, but low in resolution estimates. Hydrological methods are considered to be most appropriate at the planning level of water resource management. Appropriate levels of EFR vary across river regimes considerably. Richter et al. (2012) propose EFR as 80% of monthly runoff as a presumptive standard, while Pastor et al. (2014) propose EFR between 25% and 46% of mean annual flow. IWMI just released a study (Sood et al., 2017) that proposes global EFR estimates for the calculation of SDG target indicators.

With the incorporation of EFR in WS indicators, threshold values between levels of WS are often chosen differently as compared to the widely-used values described by Raskin et al. (1997).

**Fig. 4:**
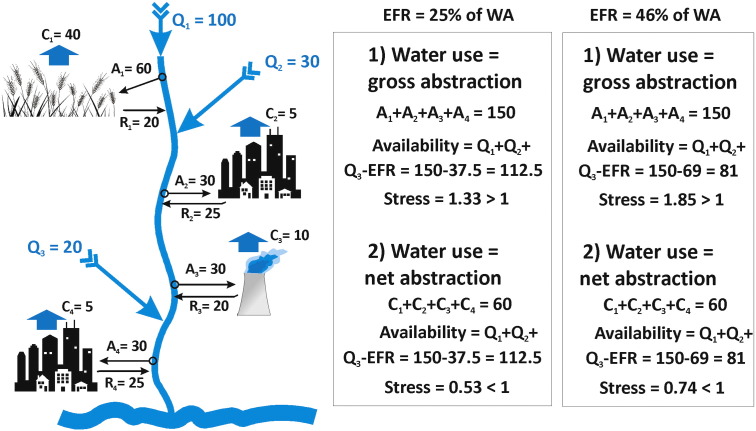
Adaptation of Fig. 2, by incorporating EFR. Two options are presented: EFR equal to 25% or 46% of water availability (WA), based upon global values listed by (Pastor et al., 2014). For both options, a clear distinction in WS quantification is seen when water use is gross or net abstraction. When EFR = 25% of WA, stress is computed to be 1.33 (larger than threshold value 1, so a situation with violation of EFR) for a gross abstraction of 150 units, whereas the stress value is 0.53 (smaller than threshold value 1, so a situation without violation of EFR) for a net abstraction of 60 units. When EFR = 46% of WA, the same observations are made but higher stress values are computed, because EFR volumes are set higher.

Fig. 4 shows an adaptation of Fig. 2, by incorporating EFR. The calculations show that: 1) WS estimates are different when computed with inclusion of EFR compared to exclusion of EFR; 2) depending on whether WS is estimated on gross or net abstractions, either WS (violation of EFR) respectively no WS (non-violation of EFR) are computed and 3) the level of EFR will result in different WS outcomes.

The importance of incorporating EFR in WS is reflected in the latest update on defining a planetary boundary on water, which now includes two definitions, one global (annual consumptive water use as blue water of 4000–6000 km^3^/yr) and a river basin scale definition based on EFR (Steffen et al., 2015). Pahl-Wostl et al. (2013) concluded that in practice, most of the approaches to quantify EFR are pragmatic and not based on ecological theory or informed analyses, due to the lack of information, in terms of flow and water use, the flow requirements of aquatic ecosystems, and the socio-economic conditions and vulnerabilities to water. Currently there is a need to estimate EFR per catchment based upon regional/local conditions and a consistent view of the desired environmental conditions.

### 2.4 Temporal scale

Both for water availability and use, there is a strong intra-annual as well as inter-annual variability.

Strong intra-annual variabilities in water availability (especially in surface water) occur in many (snow-dominated) mountain regions and their water dependent lowlands (Vanham, 2012, Viviroli et al., 2007), in monsoon-dominated river basins (Bookhagen and Burbank, 2010, Vanham et al., 2011) and other regions with distinct wet and dry periods during the year like the Mediterranean region (García-Ruiz et al., 2011), the Sahel (Aich et al., 2014) or Southern Africa (Beck and Bernauer, 2011).

Blue water use shows a high intra-annual water variability in many regions of the world as well (Veldkamp et al., 2015). Irrigation requirements depend on climatological conditions, e.g. being highest in summer in Europe (Wriedt et al., 2009). Also for EFR, it is the shape of the hydrograph over time (periods of low and high water flows) that determines ecological functions often more than annual total volumes. Annual water availability, water use and EFR amounts give no to little information on these important issues.

Water availability also has a strong inter-annual variability, as shown in the occurrence of climatological/hydrological wet, normal or dry years (Vanham et al., 2009b). Especially the blue water use of crop production can show inter-annual variability due to climatological conditions.

In the past, most WS assessments were conducted with an annual time step, e.g. Arnell (1999), Arnell (2004), Oki et al. (2001), Seckler et al. (1999) or Vörösmarty et al. (2000), thereby neglecting the high temporal variability in water use and availability that exists in most regions of the world. A smaller time step is recommended, based upon the geographical setting and scope of a study. For global assessments, a monthly time step is recommended, as recently conducted by different authors (Hoekstra et al., 2012, Mekonnen and Hoekstra, 2016, Wada et al., 2011). Also more regional assessments have been conducted with a monthly time step, e.g. Fasel et al. (2016), Milano et al. (2015) or Schyns and Hoekstra (2014).

### 2.5 Spatial resolution

In the past, global WS assessments have been made on the national level (Arnell, 1999, Oki et al., 2001, Vörösmarty et al., 2000), watershed level (Arnell, 2004, Hoekstra et al., 2012), food production unit level (a combination of watersheds and administrative boundaries) (Kummu et al., 2010, Veldkamp et al., 2015) and grid level (Arnell, 2004, Mekonnen and Hoekstra, 2016, Vörösmarty et al., 2000, Wada et al., 2011), with in the latter case often re-aggregation to (sub)basin scale. The most detailed spatial resolution of global grid-based approaches is currently 30 arc-minute (0.5° or about 55 km at the equator). Main restricting factors to the resolution of such assessments are data availability and computation time. Data on water use in global crop production is an essential restricting factor, with most detailed global assessments going down to 5 arc-minute (0.0833° or about 10 km at the equator) (Liu et al., 2013, Liu and Yang, 2010, Mekonnen and Hoekstra, 2011, Wada et al., 2016).

Regional WS assessments have been conducted with much finer resolutions, based upon more detailed regional data. The grid size of a WS case study in Austria by Vanham et al. (2009b) and Vanham et al. (2009a) is e.g. 250 m, but the authors chose to aggregate the WS results to the sub-basin level. This shows that global WS assessments are generally very coarse as compared to regional assessments. In many cases aggregation to administrative boundaries is conducted, e.g. (De Roo et al., 2016).

**Fig. 5:**
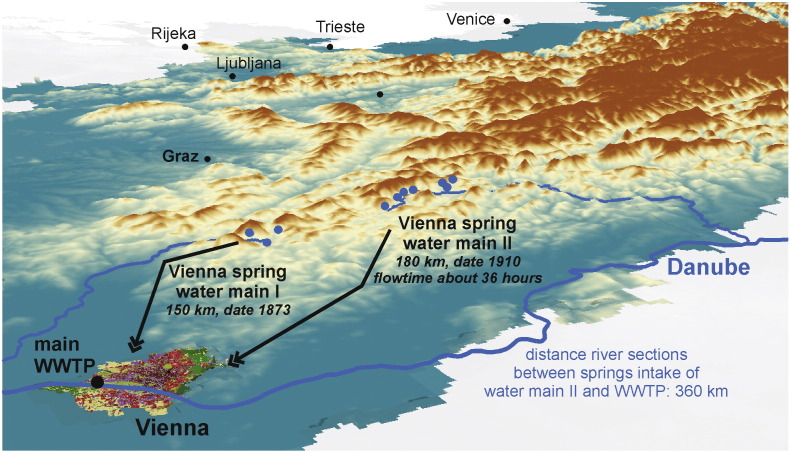
Under normal conditions, the public water supply system of Vienna is served with spring water from the Alps, supplied by two water mains, although the Danube flows through the city. Historically, this decision was made as local water quality was not good enough to serve a rapidly increasing population, leading to frequent cholera and typhus outbreaks. Vienna spring water main II abstracts water from a series of springs and conducts it in 36 h over a distance of 180 km to the city. After treatment in the main WWTP of Vienna, the return flow is released in the Danube. The river sections affected between point of abstraction and return flow measure 360 km. There is also a time difference between the two routes, as water flows more rapidly from the springs to the WWTP in the main as in the river, due to different distances but also a difference in hydraulic roughness. In monthly ES assessments this time difference will not make a difference, in short temporal analyses (e.g. daily) this makes a difference and should be accounted for. City of Vienna displayed in CORINE land cover colours.
• Surface water can be diverted from one basin to another;
• Aquifers can be transboundary over different (sub-)basins, as their extent does often not correspond to topographic basins;
• Gross water abstraction from a confined aquifer within a grid cell can originate from groundwater recharge within another cell;
• Karstic regions have very particular spatially distinct and complex groundwater recharge and discharge conditions (Malago et al., 2016). Groundwater availability can in praxis be concentrated in a spring, hence it is more meaningful to present WS at an up-scaled spatial level.

There are good reasons to aggregate grid-cell WS information to sub-basin level (Vanham et al., 2009a, Vanham et al., 2009b, Wada and Bierkens, 2014) in order to provide meaningful information:

• The distance between gross water abstraction and return flow for a water user can be substantial, and therefore not captured within a grid cell. The water supply of Vienna is an extreme example of such a situation (Fig. 5);

### 2.6 Surface water and renewable groundwater

Most existing indicators of WS compare water use with water availability (renewable water resources, i.e. surface and groundwater), with or without incorporating EFR. Groundwater is an active part of the hydrologic cycle, often closely linked to surface water features such as rivers, lakes or wetlands. But its flux, storage and residence time markedly differ from other parts of the hydrologic cycle (Aeschbach-Hertig and Gleeson, 2012).

**Fig. 6:**
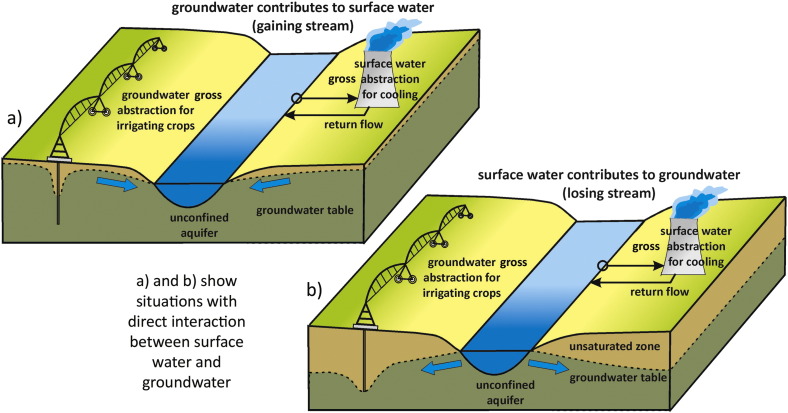
Surface water and groundwater can be in direct mutual interaction, both in gaining streams (a) or losing streams (b).

Surface water and groundwater are often in direct mutual interaction (Fig. 6) (Winter et al., 1999). In such situations, surface water use will impact groundwater resources, while groundwater use will impact river discharge. Surface and groundwater use are therefore influencing and visible in river flows (river flow measurements). Potential EFR violations in a WS assessment are thereby the result of both surface water and groundwater use. In most existing WS assessments, a differentiation between surface water and groundwater (both use and availability) is not made. Recently some WS indicators have however been developed with the aim to differentiate. Gleeson et al. (2012) e.g. developed a method to compute groundwater scarcity, based upon abstraction of groundwater, recharge rate, and the groundwater contribution to environmental streamflow.

**Fig. 7:**
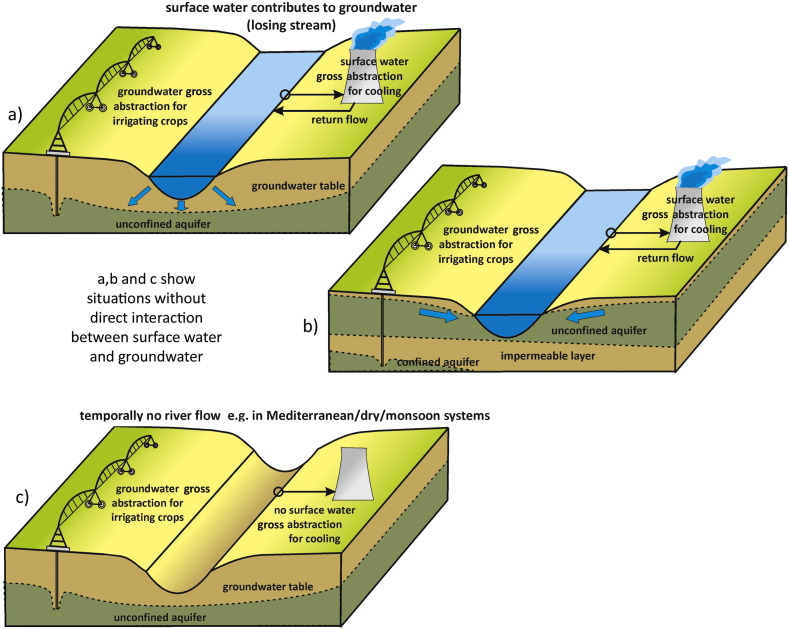
Selected situations where surface and groundwater are not in direct mutual interaction with each other. In a) surface water contributes to the unconfined aquifer below the river bed, without direct interaction. In b) water for irrigation is abstracted from a confined aquifer, which has no direct interaction with the surface water. In c) there is an intermittent river which flows part of the year, e.g. like in Mediterranean or monsoonal river systems where during the dry season rivers can naturally run dry.

There are however also situations where surface water and groundwater are indirectly connected, (temporally) disconnected, or where deeper groundwater is not connected to shallow groundwater or surface water. Fig. 7 shows three such situations, where, as a result of this small or absence of interaction, groundwater use is not affecting and therefore not represented in local river flows. When water availability in a WS assessment is based upon river flow measurements, the decrease in these groundwater stocks will not be accounted for.

### 2.7 Alternative water resources

**Fig. 8:**
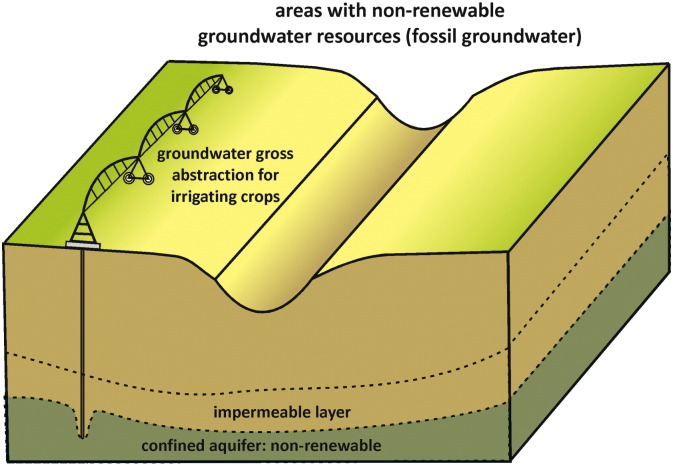
Situation where irrigation water is abstracted from fossil (non-renewable) groundwater, often at great depth. This can be in hyper-arid regions, but also in arid or semi-arid regions that have some recharge (a few mm/yr), which is much less than abstraction. In this case, deeper groundwater stocks are being mined, with differing degrees of strategic planning and efficiency of utilization.

It has appeared to be difficult how to account for alternative water resources (or water availability) in WS assessments. This holds for both fossil groundwater (Scanlon et al., 2012) (Fig. 8) and water from desalination. Increasingly desalination provides water availability (Ghaffour et al., 2013). Wada et al. (2011) accounted in their WS assessment for fossil groundwater and desalination by subtracting the volume of desalinated water and abstracted non-renewable groundwater from the water demand prior to the calculation of WS. This is the proper way to get a picture of the intensity of use of the available renewable freshwater resources, but does not provide information on the rate of fossil groundwater depletion. This needs to be looked at separately, in addition to the degree of renewable water resources appropriation.

### 2.8 Reservoirs, water recycling and managed aquifer recharge

Due to high spatial and temporal variability in water availability and water use, a large number of reservoirs has been constructed worldwide (Liu et al., 2015). Many of them are also used for hydropower generation. The timing of water use from water stored in reservoirs is different from the timing of water directly taken from the environment. In order to compute WS, reservoirs have to be included. Additionally, water evaporation from reservoirs should be accounted as water use. In many existing WS assessments, reservoirs are included, e.g. Wada et al. (2011). Faergemann (2012) also indicates that for the indicator WEI +, water availability includes storage (natural lakes but also artificial reservoirs). EFR however need to be quantified based upon pristine or naturalized river flows, i.e. the situation before the construction of man-made reservoirs.

Water is increasingly recycled (Simons et al., 2015). It is used again for the same process or activity or reused, i.e. used again but for another process or activity. Water recycling or water reuse in itself does not mean that less water is used or consumed in a process or activity. It means that less water needs to be abstracted from groundwater or surface water, but also that the return flow is smaller. Recycling or reuse of water does not reduce total net water abstraction in a catchment and in this sense not reduce water stress in the catchment (Hoekstra et al., 2011). Recycling or reuse of water, however, may provide greater reliability of water supply for the users having access to recycled or reuse water. It does have an effect when WS is computed with gross abstractions.

Managed aquifer recharge (MAR) has become a major form of intervention in many basins (Dillon et al., 2009). It is applied for a host of reasons: increasing groundwater storage, improving the quality of saline groundwater, protecting aquifers from seawater intrusion, balancing-out the mismatch between water supply and demand over short and long time-scales, enhancing river base flow and improving the quality of wastewater prior to use (Dillon et al., 2009). An emerging inventory of MAR schemes reveals about 1200 cases from 62 countries (Stefan and Ansems, 2016). This is likely to underestimate the level of actual MAR applications in many regions. The demonstrated role of MAR in adapting to climate variability and global change indicates that its importance will increase over time. As MAR alters the temporality of water availability, by storing water underground, it should be accounted for in WS assessments, in a comparable way as surface water reservoirs.

## 3 SDG indicator 6.4.2: considerations and recommendations

### 3.1 Introduction

Here we analyse the definition, concept and method of SDG indicator 6.4.2 (Section 3.2). In Section 3.3, we then analyse whether the 7 discussed elements are represented in SDG indicator 6.4.2, highlighting current shortcomings and recommendations for improvement. Section 3.4 provides with Table 6 an overview.

### 3.2 General description of indicator 6.4.2

SDG indicator 6.4.2 measures the level of water stress (WS), as computed in Eq. (1) (Table 1). Following the definitions of AQUASTAT, water withdrawal is synonymous with water abstraction. The Food and Agricultural Organisation (FAO) of the United Nations (UN) is responsible for this indicator. The indicator provides an estimate of pressure by all sectors on a country's renewable freshwater resources (FAO, 2017):

• A low level of WS indicates a situation where the combined withdrawal by all sectors is marginal in relation to the resources, and has therefore little potential impact on the sustainability of the resources or on the potential competition between users (FAO, 2017)
• A high level of WS indicates a situation where the combined withdrawal by all sectors represents a substantial share of the total renewable freshwater resources, with potentially larger impacts on the sustainability of the resources and potential situations of conflicts and competition between users (FAO, 2017). A high level of WS can result in negative effects on economic development

Total renewable freshwater resources (Table 1, TRWR) are expressed as the sum of internal renewable water resources (IRWR) and external renewable water resources (ERWR). The term “water resources” is understood as freshwater resources (FAO, 2017):

• IRWR is defined as the long-term average annual flow of rivers and recharge of groundwater for a given country generated from endogenous precipitation.
• ERWR refers to the flows of water entering the country, taking into consideration the quantity of flows reserved to upstream and downstream countries through agreements or treaties (and, where applicable, the reduction of flow due to upstream withdrawal).

Total freshwater withdrawal (Table 1, WW) is the volume of freshwater extracted from its source (rivers, lakes, aquifers) for all economic activities (based on ISIC categories, version 4) (UN, 2017). It is estimated at the country level for the following three main sectors: agriculture (includes water withdrawn for irrigation, livestock and aquaculture purposes), municipalities (including domestic water withdrawal) and industries (including cooling of thermoelectric plants) (FAO, 2017). Freshwater withdrawal includes primary freshwater (water not withdrawn before), secondary freshwater (water previously withdrawn and returned to rivers and groundwater, such as discharged treated wastewater and discharged agricultural drainage water) and fossil groundwater. It does not include direct use of non-conventional water, i.e. direct use of treated wastewater, direct use of agricultural drainage water and use of desalinated water.

Environmental flow requirements (EFR) are the quantities of water required to sustain freshwater and estuarine ecosystems. Water quality and also the resulting ecosystem services are excluded from this formulation which is confined to water volumes (water quantity).

By including EFR in the calculation of the indicator, in principle no environmental water scarcity should be considered up to a value of the indicator of 100%. However, from the perspective of water usage for human needs, there are forms of water utilization, such as navigation or recreation, which do not imply withdrawal but still require a water flow beyond the EFR. Hence, it is proposed to consider serious water scarcity at 70% as indicator's value (FAO, 2017). It is acknowledged that resulting stress values (Eq. (1), Table 1) can exceed 100%, e.g. when EFR is violated or renewable groundwater is over-abstracted.

The data for this indicator should be collected annually (FAO, 2017). However, according to the same document, a reporting period up to three years can still be considered acceptable. Within the SDG process, the indicator has to be reported at country level. Nonetheless, data collection at sub-national level would be advisable wherever possible, as that would provide a kind of information much more useful for decision making and implementation of water management plans. The disaggregation of the information at sub-national level should be done by basin units, collecting the data at the relevant level and considering the possible artificial transfer of water between basins. Different monitoring levels are distinguished for 6.4.2, recognizing that countries have different starting points when it comes to water stress monitoring, and allowing countries to begin monitoring efforts at a level in line with their national capacity and available resources, and from there advance progressively (Table 4).

**Table 4:** Monitoring ladder with proposed levels, according to (FAO, 2017).

1st step of progressive monitoring	2nd step of progressive monitoring	3rd step of progressive monitoring
The indicator can be populated with estimations based on national data aggregated to the country level. If needed, data can be retrieved from internationally available databases on water availability and withdrawals by different sectors. Inclusion of estimation of EFR based on literature values.	The indicator can be populated with nationally produced data, which increasingly can be disaggregated to the sub-national basin unit level. Inclusion of estimation of EFR based on literature values.	For more advanced levels, the nationally produced data have high spatial and temporal resolution (e.g. geo-referenced and based on metered volumes) and can be fully disaggregated by source (surface water/groundwater) and use (economic activity). Literature values of EFR are refined by national estimations.

### 3.3 Does indicator 6.4.2 consider the different elements identified in Section 2?

#### 3.3.1 Gross versus net water abstraction

In the current definition of SDG indicator 6.4.2 (Eq. (1) and Eq. (2)), water use is interpreted as gross “withdrawal” or gross abstraction. We argue that both gross and net abstraction provide important information to understand WS. As follows from Fig. 2, WS computed with gross abstraction generally overestimates actual biophysical WS. Therefore, we recommend to estimate WS according to indicator 6.4.2 based on both gross and net water abstraction (resulting in two different WS values).

#### 3.3.2 Environmental flow requirements (EFR)

The fact that SDG indicator 6.4.2 includes EFR is a very positive and essential development from the Millennium Development Goal (MDG) 7.5 indicator on WS “*proportion of total water resources used*”, since the latter did not account for EFR.

In its current definition (FAO, 2017), it is proposed to take 70% as the threshold value to indicate severe water stress, instead of 100% (see Section 2). This is debatable.

We showed that the choice of EFR amounts has an important impact on the quantification of WS (Fig. 4). The use of catchment-specific quantification of EFR, as recommended at the most advanced of monitoring (Table 4), is to be supported. A guidance document was delivered by Sood et al. (2017).

Due to the incorporation of EFR in SDG indicator 6.4.2, this indicator is referred to as a “multipurpose indicator” in the specific description of SDG targets and indicators. It can therefore also be used to report on the other targets 6.6 and 15.1 (Table 5).

**Table 5:** Other SDG targets for which indicator 6.4.2 provides relevant information.

Target	Indicator
6.6: By 2020, protect and restore water-related ecosystems, including mountains, forests, wetlands, rivers, aquifers and lakes	6.6.1: Change in the extent of water-related ecosystems over time
15.1: By 2020, ensure the conservation, restoration and sustainable use of terrestrial and inland freshwater ecosystems and their services, in particular forests, wetlands, mountains and drylands, in line with obligations under international agreements	15.1.2: Proportion of important sites for terrestrial and freshwater biodiversity that are covered by protected areas, by ecosystem type

#### 3.3.3 Temporal scale

The first two levels in the proposed monitoring ladder (Table 4) call for annual WS values. This results in a limited assessment of WS. In the advanced level 3 of the monitoring ladder, a high temporal resolution is required (FAO, 2017). Monthly assessments are state of the art. Both (annual and monthly) can also be used in parallel, displaying different things complementing each other. This is recommended for indicator 6.4.2.

As the definition of SDG target 6.4 includes to “*substantially reduce the number of people suffering from water scarcity*”, it is important how to quantify this number. Indeed, by quantifying WS on a monthly level, Mekonnen and Hoekstra (2016) come to the high amount of 4.0 billion people that live under conditions of severe water scarcity at least 1 month of the year. The number reduces to half a billion all year round, which shows the importance of the temporal scale.

To compensate for inter-annual variations in water availability, FAO's current recommendation is to use long-term average values (Section 3.2). Water use however represents a value for the reporting year. Our recommendation is to compute WS per year based on year-specific data for both water use and water availability when data availability allows.

#### 3.3.4 Spatial resolution

The first level in the proposed monitoring ladder (Table 4) calls for national WS values. The second level calls for disaggregation to the sub-national basin unit level. At the advanced level 3, a high spatial resolution is required. The recommendations of Section 2.5 need to be taken into account. Also in FAO (2017) it is stated that the possible artificial transfer of water between basins needs to be considered.

Again, as the definition of SDG target 6.4 includes to “*substantially reduce the number of people sufferinge from water scarcity*”, it is important how to quantify the number of people. This number depends on the level of spatial detail. It is recommended here that WS is estimated at different spatial scales, because WS at grid-scale can disclose high local levels of WS that would be hidden in an assessment at the scale of a catchment or nation, but WS at catchment or national level can show the broader picture for a larger area that is useful for inter-basin or international comparisons.

#### 3.3.5 Surface water and renewable groundwater

In the definition of available renewable water resources, a differentiation between surface water and groundwater is made. In the advanced level 3 of the monitoring ladder (Table 4), WS can be fully disaggregated by source (surface water and groundwater) and use (economic activity) (FAO, 2017). For the first two levels, this disaggregation is not required. For level 3, we recommend to differentiate also between renewable and non-renewable groundwater use. At level 3, three different WS estimations are to be made: a first WS estimate based on the sum of water use from renewable groundwater and surface water (to be compared to the total renewable water resources); a second WS estimate focused on renewable groundwater use versus groundwater recharge; and a third WS estimate by considering the depletion rate for non-renewable groundwater.

#### 3.3.6 Alternative water resources

As discussed in Section 3.2, gross freshwater abstraction in the equation of SDG indicator 6.4.2 (Eq. (1), Table 1) includes fossil groundwater. Fossil groundwater however is not included in water availability, as water availability only refers to renewable water in Eq. (1). This is inconsistent; by considering fossil groundwater use as a claim on the renewable water resources (while it is not), scarcity of the renewable water resources is overestimated. On the other hand, possible depletion of fossil groundwater is not made explicit by comparing fossil groundwater use to renewable water resources rather than to the available groundwater stock.

Desalinated water is subtracted from total gross freshwater abstraction in Eq. (1). Desalinated water is not included as available water resource in Eq. (1).

We recommend the approach of Wada et al. (2011), who subtracted the volumes of desalinated water and non-renewable groundwater from the water demand prior to the calculation of WS. This, however, introduces the need to consider depletion of non-renewable groundwater resources separately, in addition to considering WS related to renewable water resources.

Desalination requires a lot of energy, thereby being an important issue in the water-energy-food (WEF) or water-energy-food-ecosystem (WEFE) nexus (Vanham, 2016). The strength of the SDG indicator framework, is that it catches a lot of trade-offs. SDG goal 7 handles energy security. However, none of its four indicators specifically incorporate desalination within energy production. Only indicator 7.3.1 “*Energy intensity measured in terms of primary energy and GDP*” indirectly captures an increase in energy use due to the use of desalinated water. When disaggregation of energy intensity by sector or industry is quantified as envisaged, energy use due to desalination can be identified.

#### 3.3.7 Reservoirs, water recycling and managed aquifer recharge (MAR)

In the document (FAO, 2017), the topics of water storage through reservoirs and MAR are not discussed. We recommend that both need to be accounted for in indicator 6.4.2. Additionally, water evaporation from reservoirs should be accounted as water use.

As discussed in Section 3.2, direct use of wastewater is subtracted from total gross water abstraction in Eq. (1) (Table 1). This is appropriate, because full recycling of water (e.g. within a factory) does not affect the water system in any way. Only when not all water used is recycled, there will be water abstraction to cover for the losses; this water abstraction will be accounted for.

### 3.4 Overview of considerations and recommendations

To summarize, we present in Table 6 an overview of the elements that need to be accounted for in a water stress indicator and indicate considerations and recommendations for SDG indicator 6.4.2.

**Table 6:** Overview of 7 key aspects that need to be considered for a WS indicator, with considerations and recommendations for SDG indicator 6.4.2.

Aspect	Description	Justification	Considerations for SDG indicator 6.4.2	Recommendations for SDG indicator 6.4.2
Gross versus net water abstraction	• Both gross and net water abstraction (withdrawal) provide important information to understand WS and therefore can be used in a WS indicator. • The use of gross and/or net water abstraction in a WS assessment depends on the scale and aim of the study. • Both methods can be used in parallel	• WS computed with gross or net water abstraction gives different results (Fig. 2, Fig. 4). • WS computed with net water withdrawal, represents the actual biophysical situation for a catchment as a whole, but underestimates WS for specific river sections between points of gross water abstraction and return flow. • WS computed with gross water abstraction overestimates the severity of the actual biophysical situation at basin level. • Gross water abstraction is for certain economic activities a determining factor • Gross water abstraction is very relevant for groundwater • In water footprint assessments, net water abstraction is used	• In the current definition of SDG indicator 6.4.2 (Eq. (1) and Eq. (2)), water use is taken as gross water “withdrawal” or abstraction.	• Calculate WS according to indicator 6.4.2 based on both gross and net water abstraction (resulting in two different WS estimates).
Environmental flow requirements (EFR)	• Including EFR is a necessity • EFR estimates are context dependent, varying across river regimes, and depending what aspects of aquatic ecosystems or ecosystem services are selected to be protected • There is a need to quantify local-specific EFR more in detail to use in WS assessments.	• EFR maintain a range of ecosystem services (ES) that depend on these flows and which contribute to specific SDGs (Fig. 3) • By including EFR, the most important WS threshold value becomes 1, as indicating violation of EFR or not. • WS estimates depend on the choice of EFR in a WS assessment, as shown in Fig. 4	• Within the definition of SDG indicator 6.4.2, EFR are included	• The inclusion of EFR in indicator 6.4.2 is as recommended • The use of catchment-specific EFR quantifications is to be supported (the most advanced level of monitoring, see Table 4).
Temporal scale and spatial resolution	• Monthly time steps are recommended for WS assessments • The spatial resolution of WS assessments depends on data availability and computation time, but also on the scope of the study • The re-aggregation of WS information on the grid level to (sub)basins or administrative boundaries may be required.	• The high temporal variability in water use and availability in most regions of the world requires a more temporal disaggregated time step than annually • There has been great progress in increasing the spatial and temporal resolution of global and regional WS assessments, due to increased data availabilities and sophisticated modelling frameworks	• Temporal scale: The first two levels in the proposed monitoring ladder (Table 4) call for annual WS values, resulting in a limited assessment of WS. At the advanced level 3 of the monitoring ladder, a high temporal resolution is required. Monthly assessments are state of the art. • Spatial resolution: The first level in the proposed monitoring ladder calls for national WS values. The second level calls for disaggregation to the sub-national basin unit level. At the advanced level 3, a high spatial resolution is required.	• Annual and monthly WS estimates can be used in parallel, displaying different things complementing each other. • It is recommended that WS is estimated at different spatial scales, because WS at grid-scale can disclose high local levels of WS that would be hidden in an assessment at the scale of a catchment or nation, but WS at catchment or national level can show the broader picture for a larger area that is useful too for inter-basin or international comparisons.
Surface water and groundwater	• Regarding renewable water availability, both surface water and groundwater need to be accounted for, where special attestation needs to be given to the fact that certain groundwater stocks – especially when not in mutual interaction with surface water - are also included.	• Renewable water availability in WS assessments includes surface water and groundwater, which can be in mutual interaction or not (Fig. 6, Fig. 7).	• At the advanced level 3 of the monitoring ladder, WS can be disaggregated by source (surface water and groundwater) and use (economic activity). • For the first two levels, this disaggregation is not required.	• At levels 1 and 2, WS is to be computed based on the sum of water use from renewable groundwater and surface water. • At level 3, three different WS estimations are to be made: a first WS estimate based on the sum of water use from renewable groundwater and surface water; a second WS estimate focused on renewable groundwater use versus groundwater recharge; and a third WS estimate by considering the depletion rate for non-renewable groundwater
Alternative water sources	• Both fossil water and desalinated water are important alternative water resources.	• The use of fossil groundwater will not affect the renewable groundwater flow, but result in the depletion of the fossil groundwater stock. • The use of desalinated water will decrease WS, but implies increased energy demand.	• Gross freshwater abstraction in Eq. (1) (Table 1) includes fossil groundwater. Water from desalination is subtracted from gross abstraction in Eq. (1)	• Estimate WS related to renewable water resources by subtracting the use of desalinated water and non-renewable groundwater from water use prior to the calculation of WS. • Estimate rate of depletion of non-renewable groundwater separately, in addition.
Reservoirs, water recycling and MAR	• The temporal storage of water in surface reservoirs or underground through MAR, results in a more even spread of water availability over time. • Water recycling or reuse can be beneficial for various reasons, but does not increase water availability.	• These three infrastructure measures/processes have increasingly been constructed or applied worldwide	• In the definition of indicator 6.4.2 (Eq. (1)), direct use of treated wastewater is subtracted from total gross water abstraction • The topics of reservoirs and MAR are not discussed in (FAO, 2017)	• Surface water storage through reservoirs and groundwater storage through MAR need to be accounted for. • Evaporation from reservoirs should be included as water use. • EFR need to be based upon natural conditions, i.e. the situation without man-made reservoirs

## 4 Monitoring levels and related data availability

The monitoring ladder methodology for indicator 6.4.2 with different levels as displayed in Table 4, has the advantage that countries can begin monitoring efforts at a level in line with their national capacity and available resources. However, it has the disadvantage that WS quantifications for different levels are not directly comparable – because of other boundary conditions like different EFR, different temporal scales or spatial resolutions.

A description on sources of data can be found in FAO (2017). In order to monitor the indicator over the years, a national data collection process needs to be established in each country. The report also discusses a step-by-step data collection process.

Six Proof of Concept countries, including the Netherlands, were invited to test the methods developed by UN organizations and to collect data for the indicators linked to SDGs 6.3 to 6.6. For more advanced levels in the monitoring ladder, it is argued by Statistics Netherlands (Graveland et al., 2016) that additional options need to be taken into account, i.e. to incorporate data from modelling and remote sensing. In their document, pros and cons of using national statistical data, remote sensing data and modelling data are listed. Remote sensing data e.g. prove to be a valuable resource for computing agricultural net water abstraction (Karimi and Bastiaanssen, 2015, Karimi et al., 2013a, Karimi et al., 2013b), with the disadvantage that it gives no direct indication on gross water abstraction (which is required in the current definition of SDG indicator 6.4.2).

## 5 Additional issues: water quality and the connection blue-green water

### 5.1 Water quality

Water quality or water pollution is rarely regarded as an important factor in a WS assessment (Vorosmarty et al., 2010). However, water pollution has become a key factor influencing sustainable development in many countries (Zeng et al., 2013), especially in developing and transition countries. Therefore, some authors developed methods to assess WS by considering both water quantity and quality, e.g. Zeng et al. (2013) and Liu et al. (2016).

SDG target 6.4 does not refer to water quality, but water quality is taken into account in SDG 6.3, which is measured by two indicators (Table 7). Target 6.3 sets out to improve ambient water quality, which is essential to protect both ecosystem health (target 6.6, Table 5) and human health, by eliminating, minimizing and significantly reducing different streams of pollution into water bodies. The main sources of pollution include wastewater from households, commercial establishments and industries (point sources), as well as runoff and groundwater infiltration from urban and agricultural land (diffuse sources). Point source pollution is especially abundant in developing and transition countries due to a lack of wastewater collection and treatment infrastructure (Laghari et al., 2012, Vanham et al., 2011). Developed nations have generally invested strongly in such infrastructure. Diffuse pollution is still abundant in developing, transition and developed countries (Bouraoui and Grizzetti, 2011, Bowes et al., 2005, Grizzetti et al., 2012, Gunkel et al., 2007).

**Table 7:** SDG target 6.3 with relevant indicators, within SDG 6 “clean water and sanitation”.

Target	Indicator
6.3: By 2030, improve water quality by reducing pollution, eliminating dumping and minimizing release of hazardous chemicals and materials, halving the proportion of untreated wastewater and substantially increasing recycling and safe reuse globally	6.3.1: Proportion of wastewater safely treated
	6.3.2: Proportion of bodies of water with good ambient water quality

Indicator 6.3.1 is defined as the percentage of wastewater generated by households (sewage and faecal sludge) and economic activities (based on ISIC categories) that is safely treated. Diffuse pollution (e.g. runoff from agriculture) will be indirectly captured by indicator 6.3.2. “Good” in the definition of indicator 6.3.2 indicates an ambient water quality that does not damage ecosystem function and human health according to core ambient water quality parameters. This indicator gives an overall picture of all pollution (including from diffuse sources not captured in indicator 6.3.1) and pollution reduction activities, and is essential to describe the environmental status of freshwater systems (feeding into indicator 6.6.1, Table 5).

Water scarcity in the sense of water quality degradation is thereby indirectly captured by these two indicators. An improvement in indicators 6.3.1 and 6.3.2 will lead to less water scarcity in the sense of water pollution.

### 5.2 The connection blue-green water

As discussed in Section 1, by focusing on blue WS, indicator 6.4.2 neither addresses green water scarcity nor green-blue water scarcity. Relevant for WS assessments, however, is the connection blue-green water, as the amount of blue water in a river basin is determined by upstream flows of green water (Karimi et al., 2013a), where:

1) The amount of green water use/flow is determined by terrestrial ecosystem functions or natural land use (e.g. forests or natural grasslands) and by consumptive water use in rainfed agriculture. Changing land uses upstream affects related green water flows and thereby downstream blue water availability.
2) Moisture feedback from green water flow in one time period contributes to generate rainfall in the next period, i.e., the green water flow in an area partially maintains local rainfall and thus blue water availability as well.

It is important to distinguish between blue and green water consumption, because opportunity costs of both types of water consumption generally differ. Besides, alteration of green water flows (upstream) typically induces shifts in blue water availability (downstream) (Gerten et al., 2015).

## 6 Conclusions

The 17 Sustainable Development Goals (SDGs) comprise 169 targets and are monitored by means of 230 individual indicators, one of which indicator 6.4.2. Like all indicators, indicator 6.4.2 is just one part of the bigger picture, providing one particular piece of information on the path to sustainable development. It quantifies blue WS. It does not give information on green water scarcity, green-blue water scarcity or economic water scarcity. In its current definition, it does not include any information on water quality, although different authors state that water quality is an integral part of WS. Nevertheless, the SDG indicator framework tackles this issue indirectly by means of other indicators, more particularly indicators 6.3.1 and 6.3.2.

We have identified seven elements that are essential when using or developing a particular WS indicator, which compares blue water use with blue water availability. By analysing how indicator 6.4.2 considers the seven elements, we see some good developments as compared to the MDG indicators. We also highlight some current shortcomings and recommendations for improvement.

We recommend that both gross and net water abstraction are used in parallel for indicator 6.4.2. Data availability for gross water abstraction may be more reliable for different water users like urban and industrial water use. However, due to developments in remote sensing and modelling, data availability for agriculture – the biggest global water user – for net water abstraction has increased drastically. By additionally using net water abstraction, also supply chain analyses (water footprint assessments) can be linked to SDG indicator 6.4.2 (Hoekstra et al., 2017).

The inclusion of EFR is indeed a good development from the MDG indicators. However, there is a need to use catchment-specific EFR quantifications. We show that WS values computed with different EFR quantifications are not directly comparable.

WS quantifications need to account for the strong spatial and temporal variability in water availability, water use and EFR. Therefore, we recommend to use both annual and monthly WS values in parallel. Regarding spatial resolution, assessing WS at high spatial resolution level has the advantage of identifying local WS, but additionally assessing WS at catchment or national level can be useful as well.

Renewable water availability in WS assessments includes surface water and groundwater. Both need to be accounted for, with the need to also distinguish between use of renewable and non-renewable groundwater. Artificial surface water and groundwater storage needs to be accounted for in WS assessments as well.

Depending on the stage in the monitoring ladder, additional data resources different from national statistics need to be taken into account, i.e. modelling and remote sensing data. We observed that WS quantifications for different levels are not directly comparable due to different boundary conditions and specifications.

## References

[bb0005] Aeschbach-Hertig W., Gleeson T. (2012). Regional strategies for the accelerating global problem of groundwater depletion. Nat. Geosci..

[bb0010] Aich V. (2014). Comparing impacts of climate change on streamflow in four large African river basins. Hydrol. Earth Syst. Sci..

[bb0015] Arnell N.W. (1999). Climate change and global water resources. Glob. Environ. Chang..

[bb0020] Arnell N.W. (2004). Climate change and global water resources: SRES emissions and socio-economic scenarios. Glob. Environ. Chang..

[bb0025] Balcerski W. (1964). Javaslat a vízi létesítmények osztályozásának új alapelveire. Vízgazdálkodás: a vízügyi dolgozók lapja (Water Management).

[bb0030] Beck L., Bernauer T. (2011). How will combined changes in water demand and climate affect water availability in the Zambezi river basin?. Glob. Environ. Chang..

[bb0035] Bookhagen B., Burbank D.W. (2010). Toward a complete Himalayan hydrological budget: spatiotemporal distribution of snowmelt and rainfall and their impact on river discharge. J. Geophys. Res. Earth Surf..

[bb0040] Bouraoui F., Grizzetti B. (2011). Long term change of nutrient concentrations of rivers discharging in European seas. Sci. Total Environ..

[bb0045] Bowes M.J., Hilton J., Irons G.P., Hornby D.D. (2005). The relative contribution of sewage and diffuse phosphorus sources in the River Avon catchment, southern England: implications for nutrient management. Sci. Total Environ..

[bb0050] Chouchane H., Hoekstra A.Y., Krol M.S., Mekonnen M.M. (2015). The water footprint of Tunisia from an economic perspective. Ecol. Indic..

[bb0055] De Roo A. (2016). Modelling Water Demand and Availability Scenarios for Current and Future Land Use and Climate in the Sava River Basin.

[bb0060] Dillon P., Pavelic P., Page D., Beringen H., Ward J. (2009). Managed aquifer recharge: An Introduction.

[bb0065] EC (2015). CIS Guidance Document n°31 - Ecological Flows in the Implementation of the Water Framework Directive.

[bb0070] EC (2017). Press Release: Urban Water Atlas for Europe – a 360° View of Water Management in Cities. http://europa.eu/rapid/press-release_IP-17-1110_en.htm.

[bb0075] EEA (2003). Water Exploitation Index. http://www.eea.europa.eu/data-and-maps/indicators/water-exploitation-index.

[bb0080] EEA (2016). Website CICES Hosted by the European Environmental Agency (EEA).

[bb0085] Faergemann H. (2012). Update on Water Scarcity and Droughts Indicator Development.

[bb0090] Falkenmark M., Gunnar L. (1974). How can we cope with the water resources situation by the year 2015?. Ambio.

[bb0095] Falkenmark M. (2007). On the Verge of a New Water Scarcity: A Call for Good Governance and Human Ingenuity.

[bb0100] FAO (2017). Step-by-Step Monitoring Methodology for Indicator 6.4.2.

[bb0105] Fasel M., Bréthaut C., Rouholahnejad E., Lacayo-Emery M.A., Lehmann A. (2016). Blue water scarcity in the Black Sea catchment: identifying key actors in the water-ecosystem-energy-food nexus. Environ. Sci. Pol..

[bb0110] García-Ruiz J.M., López-Moreno J.I., Vicente-Serrano S.M., Lasanta–Martínez, T., Beguería, S. (2011). Mediterranean water resources in a global change scenario. Earth Sci. Rev..

[bb0115] Gawlik B.M. (2017). Urban Water Atlas for Europe.

[bb0120] Gerten D. (2011). Global water availability and requirements for future food production. J. Hydrometeorol..

[bb0125] Gerten D. (2015). Response to comment on “planetary boundaries: guiding human development on a changing planet”. Science.

[bb0130] Ghaffour N., Missimer T.M., Amy G.L. (2013). Technical review and evaluation of the economics of water desalination: current and future challenges for better water supply sustainability. Desalination.

[bb0135] Gleeson T., Wada Y., Bierkens M.F.P., van Beek L.P.H. (2012). Water balance of global aquifers revealed by groundwater footprint. Nature.

[bb0140] Graveland C. (2016). Sustainable Development Goals for Water - SDG 6.4 - Three Step Approach for Monitoring.

[bb0145] Grizzetti B., Bouraoui F., Aloe A. (2012). Changes of nitrogen and phosphorus loads to European seas. Glob. Chang. Biol..

[bb0150] Grizzetti B., Lanzanova D., Liquete C., Reynaud A., Cardoso A.C. (2016). Assessing water ecosystem services for water resource management. Environ. Sci. Pol..

[bb0155] Gunkel G. (2007). Sugar cane industry as a source of water pollution – case study on the situation in Ipojuca River, Pernambuco, Brazil. Water Air Soil Pollut..

[bb0160] Hirji R., Davis R. (2009). Environmental flows in water resources policies, plans, and projects. Findings and Recommendations.

[bb0165] Hoekstra A.Y., Mekonnen M.M. (2012). The water footprint of humanity. Proc. Natl. Acad. Sci..

[bb0170] Hoekstra A.Y., Chapagain A.K., Aldaya M.M., Mekonnen M.M. (2011). The Water Footprint Assessment Manual: Setting the Global Standard.

[bb0175] Hoekstra A.Y., Mekonnen M.M., Chapagain A.K., Mathews R.E., Richter B.D. (2012). Global monthly water scarcity: blue water footprints versus blue water availability. PLoS One.

[bb0180] Hoekstra A.Y., Chapagain A.K., Van Oel P.R. (2017). Advancing water footprint assessment research: challenges in monitoring progress towards sustainable development goal 6. Water.

[bb0185] ISO (2014). ISO 14046 - Environmental Management - Water Footprint - Principles, Requirements and Guidelines.

[bb0190] Karimi P., Bastiaanssen W.G.M. (2015). Spatial evapotranspiration, rainfall and land use data in water accounting; part 1: review of the accuracy of the remote sensing data. Hydrol. Earth Syst. Sci..

[bb0195] Karimi P., Bastiaanssen W.G.M., Molden D. (2013). Water accounting plus (WA +); a water accounting procedure for complex river basins based on satellite measurements. Hydrol. Earth Syst. Sci..

[bb0200] Karimi P., Bastiaanssen W.G.M., Molden D., Cheema M.J.M. (2013). Basin-wide water accounting based on remote sensing data: an application for the Indus Basin. Hydrol. Earth Syst. Sci..

[bb0205] Kummu M., Ward P.J., de Moel H., Varis O. (2010). Is physical water scarcity a new phenomenon? Global assessment of water shortage over the last two millennia. Environ. Res. Lett..

[bb0210] Kummu M., Gerten D., Heinke J., Konzmann M., Varis O. (2014). Climate-driven interannual variability of water scarcity in food production potential: a global analysis. Hydrol. Earth Syst. Sci..

[bb0215] Kummu M. (2016). The world's road to water scarcity: shortage and stress in the 20th century and pathways towards sustainability. Sci Rep.

[bb0220] Laghari A.N., Vanham D., Rauch W. (2012). The Indus basin in the framework of current and future water resources management. Hydrol. Earth Syst. Sci..

[bb0225] Liu J., Yang H. (2010). Spatially explicit assessment of global consumptive water uses in cropland: green and blue water. J. Hydrol..

[bb0230] Liu J. (2013). A global and spatially explicit assessment of climate change impacts on crop production and consumptive water use. PLoS One.

[bb0235] Liu J., Zhao D., Gerbens-Leenes P.W., Guan D. (2015). China's rising hydropower demand challenges water sector. Sci Rep.

[bb0240] Liu J., Liu Q., Yang H. (2016). Assessing water scarcity by simultaneously considering environmental flow requirements, water quantity, and water quality. Ecol. Indic..

[bb0245] Liu J. (2017). Water scarcity assessments in the past, present and future. Earth's Future.

[bb0250] Lokgariwar C., Chopra R., Smakhtin V., Bharati L., O'Keeffe J. (2014). Including cultural water requirements in environmental flow assessment: an example from the upper Ganga River, India. Water Int..

[bb0255] Malago A. (2016). Regional scale hydrologic modeling of a karst-dominant geomorphology: the case study of the island of Crete. J. Hydrol..

[bb0260] Mather R. (2009). Wetlands in the Mekong Basin.

[bb0265] Mekonnen M.M., Hoekstra A.Y. (2011). The green, blue and grey water footprint of crops and derived crop products. Hydrol. Earth Syst. Sci..

[bb0270] Mekonnen M.M., Hoekstra A.Y. (2016). Four billion people facing severe water scarcity. Sci. Adv..

[bb0275] Milano M., Reynard E., Köplin N., Weingartner R. (2015). Climatic and anthropogenic changes in Western Switzerland: impacts on water stress. Sci. Total Environ..

[bb0280] Molden (2007). Water for Food, Water for Life - A Comprehensive Assessment of Water Management in Agriculture.

[bb0285] Molle F., Wester P., Hirsch P. (2010). River basin closure: processes, implications and responses. Agric. Water Manag..

[bb0290] Munia H. (2016). Water stress in global transboundary river basins: significance of upstream water use on downstream stress. Environ. Res. Lett..

[bb0295] Oki T. (2001). Global assessment of current water resources using total runoff integrating pathways. Hydrol. Sci. J..

[bb0300] Pahl-Wostl C. (2013). Environmental flows and water governance: managing sustainable water uses. Curr. Opin. Environ. Sustain..

[bb0305] Pastor A.V., Ludwig F., Biemans H., Hoff H., Kabat P. (2014). Accounting for environmental flow requirements in global water assessments. Hydrol. Earth Syst. Sci..

[bb0310] Raskin P., Gleick P., Kirshen P., Pontius G., Strzepek K. (1997). Water futures: assessment of long-range patterns and problems. Comprehensive Assessment of the Freshwater Resources of the World.

[bb0315] Richter B.D., Davis M.M., Apse C., Konrad C. (2012). A presumptive standard for environmental flow protection. River Res. Appl..

[bb0320] Rijsberman F.R. (2006). Water scarcity: fact or fiction?. Agric. Water Manag..

[bb0325] Rockström J. (2009). Future water availability for global food production: the potential of green water for increasing resilience to global change. Water Resour. Res..

[bb0330] Savenije H.H.G. (2000). Water scarcity indicators; the deception of the numbers. Phys. Chem. Earth Part B.

[bb0335] Scanlon B.R. (2012). Groundwater depletion and sustainability of irrigation in the US High Plains and Central Valley. Proc. Natl. Acad. Sci..

[bb0340] Schyns J.F., Hoekstra A.Y. (2014). The added value of water footprint assessment for national water policy: a case study for Morocco. PLoS One.

[bb0345] Schyns J.F., Hamaideh A., Hoekstra A.Y., Mekonnen M.M., Schyns M. (2015). Mitigating the risk of extreme water scarcity and dependency: the case of Jordan. Water Int..

[bb0350] Schyns J.F., Hoekstra A.Y., Booij M.J. (2015). Review and classification of indicators of green water availability and scarcity. Hydrol. Earth Syst. Sci..

[bb0355] Seckler D., Barker R., Amarasinghe U. (1999). Water scarcity in the twenty-first century. Int. J. Water Resour. Dev..

[bb0360] Simons G.W.H., Bastiaanssen W.G.M., Immerzeel W.W. (2015). Water reuse in river basins with multiple users: a literature review. J. Hydrol..

[bb0365] Smakhtin V., Revenga C., Döll P. (2004). Taking into Account Environmental Water Requirements in Global-Scale Water Resources Assessments.

[bb0370] Sood A. (2017). Global Environmental Flow Information for the Sustainable Development Goals.

[bb9000] Stefan C., Ansems N. (2016). Web-GIS of global inventory of managed aquifer recharge (MAR) applications. http://marportal.un-igrac.org.

[bb0375] Steffen W. (2015). Planetary boundaries: guiding human development on a changing planet. Science.

[bb0380] Szesztay K. (1970). The Hydrosphere and the Human Environment.

[bb0385] Tharme R.E. (2003). A global perspective on environmental flow assessment: emerging trends in the development and application of environmental flow methodologies for rivers. River Res. Appl..

[bb0390] UfM (2017). Press Release: UfM Ministers Agree on New Framework for an Enhanced Regional Cooperation on Water in the Mediterranean. http://ufmsecretariat.org/ufm-ministers-agree-on-new-framework-for-an-enhanced-regional-cooperation-on-water-in-the-mediterranean-2/.

[bb0395] UN (1997). Comprehensive Assessment of the Freshwater Resources of the World.

[bb0400] UN (2017). International Standard Industrial Classification of All Economic Activities. https://unstats.un.org/unsd/cr/registry/regcst.asp?Cl=27.

[bb0405] Vanham D. (2012). The Alps under climate change: implications for water management in Europe. Journal of Water and Climate Change.

[bb0410] Vanham D. (2016). Does the water footprint concept provide relevant information to address the water–food–energy–ecosystem nexus?. Ecosyst. Serv..

[bb0415] Vanham D., Bidoglio G. (2014). The water footprint of Milan. Water Sci. Technol..

[bb0420] Vanham D., Fleischhacker E., Rauch W. (2009). Impact of an extreme dry and hot summer on water supply security in an alpine region. Water Sci. Technol..

[bb0425] Vanham D., Fleischhacker E., Rauch W. (2009). Impact of snowmaking on alpine water resources management under present and climate change conditions. Water Sci. Technol..

[bb0430] Vanham D., Weingartner R., Rauch W. (2011). The Cauvery river basin in Southern India: major challenges and possible solutions in the 21st century. Water Sci. Technol..

[bb0435] Veldkamp T.I.E. (2015). Changing mechanism of global water scarcity events: impacts of socioeconomic changes and inter-annual hydro-climatic variability. Glob. Environ. Chang..

[bb0440] Viviroli D., Dürr H.H., Messerli B., Meybeck M., Weingartner R. (2007). Mountains of the world, water towers for humanity: typology, mapping, and global significance. Water Resour. Res..

[bb0445] Vörösmarty C.J., Green P., Salisbury J., Lammers R.B. (2000). Global water resources: vulnerability from climate change and population growth. Science.

[bb0450] Vorosmarty C.J. (2010). Global threats to human water security and river biodiversity. Nature.

[bb0455] Wada Y., Bierkens M.F.P. (2014). Sustainability of global water use: past reconstruction and future projections. Environ. Res. Lett..

[bb0460] Wada Y. (2011). Global monthly water stress: 2. Water demand and severity of water stress. Water Resour. Res..

[bb0465] Wada Y., de Graaf I.E.M., van Beek L.P.H. (2016). High-resolution modeling of human and climate impacts on global water resources. J. Adv. Model. Earth Syst..

[bb0470] Winter T.C., Harvey J.W., Franke O.L., Alley W.M. (1999). Ground Water and Surface Water, a Single Resource.

[bb0475] Wriedt G., Van der Velde M., Aloe A., Bouraoui F. (2009). Estimating irrigation water requirements in Europe. J. Hydrol..

[bb0480] Zeng Z., Liu J., Savenije H.H.G. (2013). A simple approach to assess water scarcity integrating water quantity and quality. Econ. Indic..

